# Late-Onset Glaucoma-Filtrating Bleb Leak in a Penetrating Keratoplasty Patient: A Case Report

**DOI:** 10.1155/2012/810751

**Published:** 2012-03-26

**Authors:** Zuleyha Yalniz-Akkaya, Ayse Burcu, Firdevs Ornek

**Affiliations:** Ophthalmolgy Clinic, Ministry of Health Ankara Training and Research Hospital, TR06340 Ankara, Turkey

## Abstract

*Introduction*. Late-onset bleb leaks occur more frequently after the use of adjunctive antimetabolites and require surgical management to seal and preserve filtrating bleb. *Case Presentation*. A 48-year-old female presented with decreased visual acuity for five days in her left eye. She had a left penetrating keratoplasty one year earlier and two trabeculectomies 7 years earlier. Visual acuity was hand motions, intraocular pressure was 3 mmHg, corneal graft was clear, mature cataract was present, and axial length was 30.48 mm. The conjunctiva covering the superotemporal sclerotomy was avascular, flat, and partially lost. After heavily painting the bleb with a fluorescein, late-onset point leak was revealed. Overlying conjunctiva was excised. The atrophic, irregular, and partially absent scleral flap was covered by a processed human pericardium graft and conjunctival advancement. Postoperatively, intraocular pressure stabilized around 16 mmHg. After four months, phacoemulsification and intraocular lens implantation were performed. Visual acuity did not exceed 0.1 (in decimal notation) due to degenerative myopia-related macular atrophy. Corneal graft remained clear at her 6-month followup period. *Conclusion*. Surgical bleb revision using a pericardium graft and conjunctival advancement seems to be an effective method for treating late bleb leaks. However, careful follow-up is required for detecting recurrent leaks and elevated intraocular pressure.

## 1. Introduction

Late-onset bleb leaks are defined as leaks occurring more than three months after trabeculectomy. Although the use of antimetabolites (mitomycin C and 5-fluorouracil) has increased the success rate of trabeculectomy, the incidence of late bleb leaks also increased along with other complications [1]. For the treatment of late bleb leaks, surgical intervention is necessary and various techniques have been described in previous reports.

## 2. Case Report

A 48-year-old female patient called to the Cornea Department prior to her appointment complaining of decreased visual acuity (VA) in her left eye for five days. She underwent left penetrating keratoplasty for nonspecific corneal leucoma one year earlier. Approximately 7 years earlier, she underwent left trabeculectomy twice, at a different institution. Data on the use of adjunctive antimetabolites was not available.

Visual acuity was measured using a Snellen chart. Intraocular pressure (IOP) was measured by a Goldmann applanation tonometer. Ocular examination was performed using a slit lamp biomicroscopy. Bleb leak was checked under cobalt blue slit-lamp illumination after staining with flourescein.

**Figure 1:**
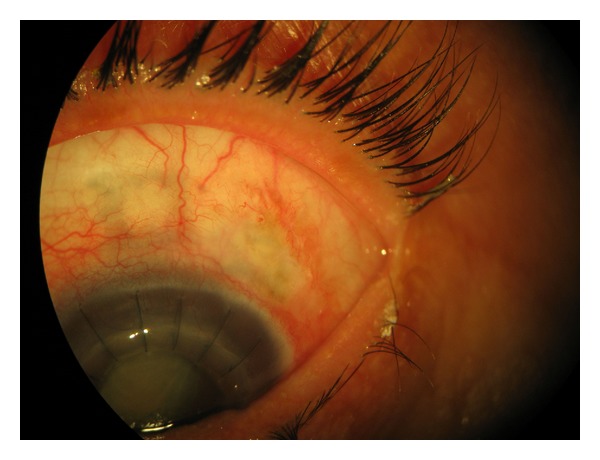
The avascular conjunctiva covering the superotemporal sclerotomy.

**Figure 2:**
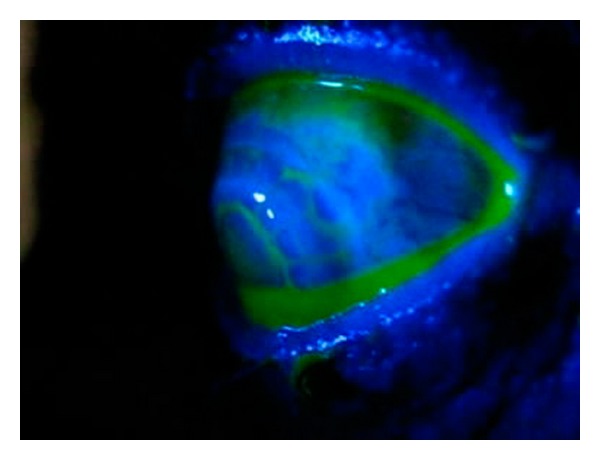
The leakage streaming down.

Over the last three months, VA decreased in her left eye from “counting fingers at one meter” to “hand motions,” the IOP decreased from 17 mmHg to 3 mmHg. The nuclear sclerosis (grade III) and posterior subcapsular cataract progressed to a mature cataract (Figure 1). Additionally, three sclerotomies (superonasally, superiorly, and superotemporally) and two iridectomies (superonasally and superotemporally) were noted. The conjunctiva covering the superotemporal sclerotomy was avascular, flat, and partially lost (Figure 1). The corneal graft was clear and secured with 16 interrupted 10-0 nylon sutures. B-scan ultrasonographic evaluation of the posterior segment was normal. Axial length was 30.48 mm. After an unsuccessful first attempt to identify a bleb leak with 2% fluorescein, we were able to observe the Siedel positivity with a heavily painted bleb and we made the diagnosis of late-onset filtrating bleb leak (Figure 2) which was a point leak but not an oozing.

In her right eye, the penetrating corneal graft was clear (for 27 years), distant corrected visual acuity was 0.5 (in decimal notation), IOP was 12 mmHg (with dorzolamide and timolol maleate combination, (Cosopt, Merck & Co. Inc., NJ, USA)). Axial length was 30.20 mm. Posterior segment was consistent with degenerative myopia.

In order to eliminate the risk of infection and obtain adequate IOP, surgical intervention was scheduled to fix the leak. The surgical procedure was performed under topical and subconjunctival anesthesia. The avascular conjunctiva was excised together with 1 mm of healthy conjunctiva. An atrophic and irregular scleral flap was exposed. The surrounding relatively healthy conjunctiva was firmly attached to the underlying sclera, causing difficult subconjunctival dissection. As the scleral flap could not be fixed properly and adequate sclera for flap creation was not available due to the firm subconjunctival scarring and degenerative myopia-associated thin sclera, the decision was made to overlay the atrophic and partially absent scleral flap with a processed human pericardium patch graft (Tutopatch, Tutogen Medical GmbH, Neunkirchen am Brand, Germany) followed by conjunctival advancement. Four interrupted 10-0 nylon (Daclon, Smi, St. Vith, Belgium), sutures were used to secure the graft. The tightness was adjusted so as to allow aqueous drainage underneath the graft and not to seal it completely. After a large subconjunctival dissection, the conjunctiva was advanced and closed with multiple 10-0 nylon sutures. The limbal side was closed with running mattress suture, and watertight wound closure was obtained with minimal tension. Ofloxacin (Exocin, Allergan Pharmaceuticals Inc., County Mayo, Ireland) and dexamethasone sodium phosphate (Maxidex, Alcon Laboratories Inc., TX, USA) were used eight times per day postoperatively for one week and one month, respectively.

**Figure 3:**
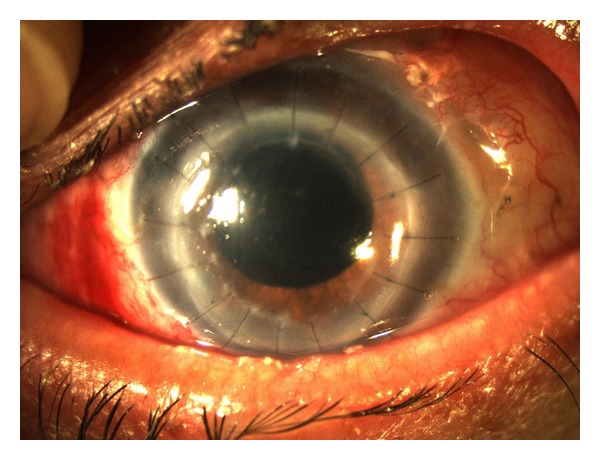
Postoperative picture: elevated filtrating bleb and cataract removed.

The IOP stabilized at 16 mmHg without medication, and a functioning filtrating bleb was formed (Figure 3). After four months, phacoemulsification and a +7.00 D intraocular lens (Acrysof MA60AC, Alcon Laboratories Inc, TX, USA) implantation were performed. Visual acuity did not exceed 0.1 (in decimal notation) due to degenerative myopia. Although macular edema was not detected after the cataract surgery, hypotony-associated macular edema sequela could have been another cause for low final visual acuity. The corneal graft remained clear at her 6-month followup, after the phacoemulsification (Figure 3).

## 3. Discussion

This is the second reported case of late bleb leak in a penetrating keratoplasty patient. The first one was a patient that developed bleb leak after combined penetrating keratoplasty and trabeculectomy with mitomycin C [2]. According to reports published to date, the prevalence of late-onset bleb leaks was reported to range between 1.4% and 14.6% with adjunctive antimetabolite application [1, 3]. Late bleb leaks can occur from three months to years after surgery; the reported mean appearance time was 27.9 months [3].

The goal of treatment is to seal the leak, eliminate the hypotony, and maintain the target IOP by preserving a functional filtering bleb. As an established treatment method is not present, several treatments have been described for the management of late bleb leaks with various success rates. Nonincisional treatments consist of observation with or without aqueous suppression, pressure patches, collagen shields, bandage contact lenses, autologous serum drops, intrableb autologous blood injections, Holmium laser bleb revision, Neodymium: Yttrium Aluminium Garnet laser bleb remodeling, Argon laser “spot-welding,” cyanoacrylate, and fibrin tissue adhesives [4]. Incisional treatments consist of conjunctival advancement or rotational flaps, free conjunctival flaps and bleb excision with amniotic membrane transplantation, patching with processed donor collagenous tissue (e.g., sclera, pericardium, or dura mater), and corneal stromal patch graft. These incisional procedures can be associated with or without bleb excision [4–7]. Incisional treatments were reported to be more effective than nonincisional treatments with respect to leak resolution [8]. The reported success rates are up to 93% [9, 10]. However, additional antiglaucomatous treatment can be required in up to 60% of patients [8–10]. After closing the trabeculectomy site with a scleral graft, Miller et al. implanted a Baerveldt Glaucoma Device to maintain drainage and eliminate the possibility of postoperative IOP rise [5].

Our patient had a leaking bleb, hypotony, hypotony-associated cataract, previous penetrating keratoplasty, and degenerative myopia. She was treated successfully with donor pericardium graft, conjunctival advancement, and cataract removal. In spite of the short-term successful treatment, corneal graft decompensation, recurrence of leakage, and elevation of IOP are potential long-term risks requiring further medical or surgical interventions.

The bleb should be routinely checked for leaks, as late-onset bleb leaks can lead to serious complications, and early diagnosis is critical for successful treatment. Fluorescein strips should be the preferred method in order to paint the bleb and reveal occult leaks.

## 4. Conclusion

Surgical bleb revision with pericardium patch graft and conjunctival advancement seems to be an effective method for treating late-onset bleb leaks. However, careful followup is required for detecting and intervening on recurrent leaks and elevated IOP.
